# Bridging knowledge to develop an action plan for integrated care for chronic diseases in Greece

**DOI:** 10.5334/ijic.2228

**Published:** 2015-10-22

**Authors:** Apostolos Tsiachristas, Christos Lionis, John Yfantopoulos

**Affiliations:** Health Economics Research Centre, Nuffield Department of Population Health, University of Oxford, UK; Clinic of Social and Family Medicine, Medical School, University of Crete, Greece; School of Economics and Political Science, University of Athens, Greece

**Keywords:** integrated care, policy reforms, chronic diseases, economic crisis, Greece

## Abstract

The health, social and economic impact of chronic diseases is well documented in Europe. However, chronic diseases threaten relatively more the ‘memorandum and peripheral’ Eurozone countries (i.e., Greece, Spain, Portugal and Ireland), which were under heavy recession after the economic crisis in 2009. Especially in Greece, where the crisis was the most severe across Europe, the austerity measures affected mainly people with chronic diseases. As a result, the urgency to tackle the threat of chronic diseases in Greece by promoting public health and providing effective chronic care while flattening the rising health care expenditure is eminent. In many European countries, integrated care is seen as a means to achieve this.

The aim of this paper was to support Greek health policy makers to develop an action plan from 2015 onwards, to integrate care by bridging local policy context and needs with knowledge and experience from other European countries. To achieve this aim, we adopted a conceptual framework developed by the World Health Organization on one hand to analyse the status of integrated care in Greece, and on the other to develop an action plan for reform. The action plan was based on an analysis of the Greek health care system regarding prerequisite conditions to integrate care, a clear understanding of its context and successful examples of integrated care from other European countries. This study showed that chronic diseases are poorly addressed in Greece and integrated care is in embryonic stage.

Greek policy makers have to realise that this is the opportunity to make substantial reforms in chronic care. Failing to reform towards integrated care would lead to the significant risk of collapse of the Greek health care system with all associated negative consequences. The action plan provided in this paper could support policy makers to make the first serious step to face this challenge. The details and specifications of the action plan can only be decided by Greek policy makers in close cooperation with other health and social care partners. This is the appropriate time for doing so.

## Introduction

Chronic diseases pose a major threat to population health and sustainability of health care systems and economies worldwide. This threat becomes higher due to the increasing prevalence of chronic diseases resulted from the rapid growth and ageing of global population accompanied by unhealthy behaviour (i.e., smoking, physical inactivity, unhealthy diet and harmful use of alcohol). Worldwide, chronic diseases cause more than 63% of all deaths, a number that is expected to increase by 15% by 2020 and by more than 20% by 2030 [1]. Chronic diseases have a high impact on global morbidity too. Seventy-seven per cent of total disability adjusted life years is caused by chronic diseases [2,3]. Furthermore, chronic diseases disproportionately affect people at lower socio-economic status because poverty exposes people to behavioural and environmental risk factors for chronic diseases and, in turn, the resulting chronic diseases may become an important driver to the downward spiral that leads families towards poverty. The social impact of chronic diseases is even larger considering their influence on mortality and quality of life of informal caregivers and family [4,5]. With regard to the economic impact, it is estimated that over the next 20 years, chronic diseases will cost more than 48% of the global gross domestic product in 2010 [6]. They account for a large extent of the increasing health care expenditure [7] because their direct and indirect costs in health care are a sizeable share of a country's gross domestic product. This share is continuously increasing due to the rising prevalence of chronic diseases [8]. As a result, chronic diseases constitute a great challenge to economies and a threat to the sustainability of health care systems worldwide. This challenge is even greater when considering that chronic diseases have long-term macroeconomic impact on consumption, capital accumulation, labour productivity and labour supply [3]. Consequently, chronic diseases jeopardise the global economic growth worldwide.

**Figure 1.**
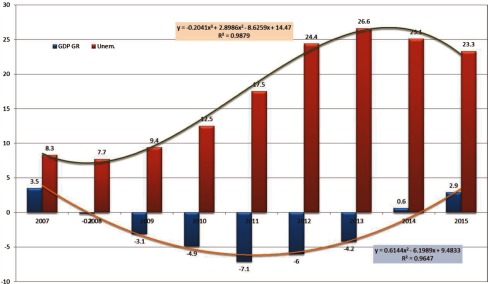
Gross domestic product growth and unemployment trends in Greece (2007–2015). Data source: World Economic Outlook Database [13]

In Europe, the health, social and economic impact of chronic diseases is well documented [3,9]. However, chronic diseases threaten relatively more the ‘memorandum and peripheral’ Eurozone countries (i.e., Greece, Spain, Portugal and Ireland), which were under heavy recession after the economic crisis in 2009. The Eurozone countries and the International Monetary Fund undertook the responsibility to provide rescue packages to Greece (on April 2010) followed by Ireland (November 2010) and Portugal (May 2011). Although public health was evidently worsened all over Europe during the economic crisis, exhaustive austerity measures in these countries resulted to heavy budget cuts and increased cost-sharing [10,11]. Especially in Greece, which has suffered from the longest and the deepest crisis in comparison to the rest ‘memorandum and peripheral’ Eurozone countries, the austerity measures affected mainly people with chronic diseases [12]. This means that the negative impact of chronic diseases on population health, health equity, health care spending and macroeconomic growth prospects was accelerated in Greece. Data from International Monetary Fund show that over the period of the last five years the Greek gross domestic product growth was reduced significantly by 29.5%, wages were reduced by 35–45%, private consumption dropped by 30% and health expenditure declined by 41%, while unemployment increased by 253% (Figure 1).

Further budget cuts without structural reforms would lead to the collapse of the health care and health protection system [14] threatening, therefore, the overall sustainability of the Greek economy. This would be undesirable for Greece and the Greek debt holders (i.e., International Monetary Fund, European Union and European Central Bank). From a more solidary point of view, tackling the increased threat of chronic diseases in Greece would partially reduce the evident health inequalities among Europeans [15]. Therefore, the urgency to tackle the threat that chronic diseases pose in Greece by promoting public health and providing effective chronic care while flattening the rising health care expenditure is eminent [16,17].

**Figure 2.**
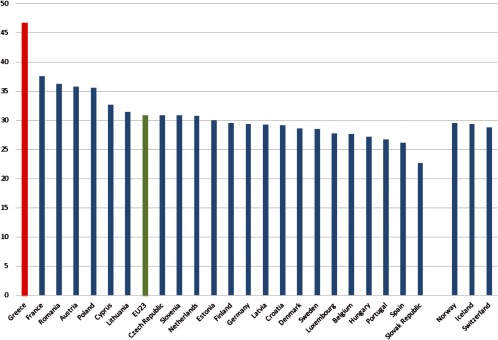
Percentage of total health expenditure spent on hospital care: Year 2012 or nearest year. Source: OECD Health Statistics 2015 [21]

Many (western) European countries recognised that the sustainability of health care systems urges the switch of care for people with chronic diseases from acute and reactive to preventive and proactive. In other words, care for people with chronic diseases requires an integrated, multidisciplinary package of well-coordinated care that includes (besides prevention) monitoring and active participation of people [18]. However, most European health care systems are currently focusing on acute, mono-disciplinary, episodic and segmented care. Therefore, many health care authorities across Europe have experimented with models for integrating care for chronic diseases. However, the Greek health care system remains conventional, pyramid-based and hospital-oriented, without multidisciplinary collaboration in clinical practice, while integrated care remains a neglected subject in the current health policy agenda in Greece [17,19,20]. According to the Organization for Economic Cooperation and Development statistics (2014), around 47% of the Greek health expenditure is devoted to hospital care, whereas the rest of the Southern European countries spent half of this amount (Figure 2). At the same time, Greece is the only Organization for Economic Cooperation and Development country with the lowest share on primary care. Only 22% of the Greek health expenditure is spent on primary care and the corresponding values for Spain and Portugal are 38% and 45%, respectively.

Designing and successfully implementing a health care reform towards integrated care in a relatively short-time and during a heavy economic recession is admittedly a very challenging task. This task should include a thorough diagnosis of the Greek health care system regarding prerequisite conditions to integrate care, a clear understanding of its context and successful examples (in terms of improving care within limited budgets) of integrated care from leading European countries in this field. The aim of this paper was to support Greek health policy makers to develop an action plan from 2015 onwards to integrate care by bridging local policy context and needs with knowledge and experience from other European countries. To achieve this aim, we adopted a conceptual framework developed by the World Health Organization on one hand to analyse the status of integrated care in Greece, and on the other to develop an action plan for reform [22]. This action plan could inspire policy makers in other European countries (e.g., ‘peripheral’ Eurozone and Eastern European countries) with similar challenges to take the first steps towards integrated care.

## Background information on integrated care

Integrated care is the most promising concept in redesigning care to tackle the increasing threat of chronic diseases. It refers to a ‘range of approaches deployed to increase coordination, cooperation, continuity, collaboration, and networking across different components of health service delivery’ [18]. It puts the patient and his or her individual needs in the centre and organises care around the patient, thereby seeking to reduce redundancies and fragmentation in health care delivery [23]. However, the term integrated care is vague and confusing because there is no commonly agreed definition and because different terms (such as coordinated care, managed care, seamless care) have been used interchangeably with integrated care [24]. The underlying denominator of integrated care definitions is the improvement of outcomes for population groups with diverse and complex needs. This is reflected in the World Health Organization's definition of integrated care, which is described as ‘a concept bringing together inputs, delivery, management and organisation of services related to diagnosis, treatment, care, rehabilitation and health promotion. Integration is a means to improve services in relation to access, quality, user satisfaction and efficiency’ [25]. Kodner and Spreeuwenberg (2002) also addressed that the aim of integrated care is ‘to enhance quality of care and quality of life, consumer satisfaction and system efficiency for patients with complex, long-term problems cutting across multiple services, providers and settings’ [24]. Busse et al. (2008) stated that integrated care aims to: (1) improve quality of care delivery, (2) ensure professional adherence to disease-specific protocols and guidelines, (3) reduce unnecessary hospital utilisation by strengthening the primary care sector, (4) share financial responsibility with other stakeholders, and in the long-term, (5) contain the increasing chronic care expenditure [26].

The first integrated care models were developed in the USA in the 1980s. Although not extended across different care sectors, the Kaiser Permanente's ‘pyramid of care’ model in California and the ‘Evercare’ model in Minnesota are the most well-known and influential ones [27]. The American experience with the identification of chronic disease and providing care according to the patients’ needs have been influential in Europe and elsewhere [28–30]. It was the basis of the introduction of case management in all Primary Care Trusts in the United Kingdom National Health System in 2007 which aimed to improve the quality and accessibility of care for people with chronic conditions and contain associated costs. In Sweden, many county councils offer chains of care for diabetes, dementia and rheumatoid arthritis [31]. In France, the formation of local provider networks for ambulatory patients was stimulated through the 2002 Patients’ Rights and Quality of Care Act [32]. Likewise, in the province of Ontario in Canada, networks of family doctors and local health integration networks were formed [33]. In many cases, the implementation of integrated care was in the form of disease management programmes [34]. Schrijvers (2009) defines these programmes as ‘a group of coherent interventions designed to prevent or manage one or more chronic conditions using a systematic, multidisciplinary approach and potentially employing multiple treatment modalities’ [35].

A systematic literature review found that the most common components of care coordination programmes were self-management support and patient education, often combined with structured clinical follow-up and case management; a multidisciplinary patient care team; multidisciplinary clinical pathways; and feedback, reminders and education for professionals [36]. Despite considerable heterogeneity in interventions, patient populations, and processes and outcomes of care, literature reviews concluded that coordination of care improves quality of care [36,37], patient's quality of life and physical exercise [38], patient self-management [39] and reduces hospitalisation [38,40]. Since the process of coordinating care effectively around the needs of people with complex needs at the clinical and service level is the most essential ingredient to integrated care (as opposed to, say, organisational mergers or new forms of shared governance that might support it), the focus of any structural reforms needs to make an impact at this level [41]. However, there is large variation in the interventions provided by integrated care programmes and the participating population, which causes large variation in their effectiveness [42]. This might be because they focus on structural and financial problems rather than coordinated activity around people's needs or a focus on getting activated patients.

## A framework to support policy reforms in chronic care

The most widespread model towards integrating care for chronic disease is Wagner's Chronic Care Model, which provides a framework of elements that must be considered when developing improvement strategies for providing care for people with chronic diseases. This originally included: (a) self-management support, (b) decision support, (c) delivery system design, (d) clinical information systems, (e) health care organisation, and (f) community resources and policies [43]. The Chronic Care Model was later extended to put more emphasis on patient safety, care coordination and case management [27]. Several studies concluded that the Chronic Care Model improves patient care and health outcomes [44,45].

Based on the Chronic Care Model, the World Health Organization developed the Innovative Care for Chronic Conditions framework to guide policy makers worldwide who are aiming to integrated care for patients with chronic diseases [22,23]. The framework is comprised of fundamental components within the patient (micro-), organisation/community (meso-) and policy (macro-) levels. These components are described as ‘building blocks’ that can be used to create or redesign a health care system that can more effectively manage chronic diseases. Decision-makers can use the building blocks to develop new systems, initiate changes in existing systems or make strategic plans for future systems [22]. The guiding principles of the Innovative Care for Chronic Conditions framework are: evidence-based decision-making, population focus, prevention focus, quality focus, integration and flexibility/adaptability. Each of these principles is crucial to the micro-, meso- and macro-level.

We adopted the Innovative Care for Chronic Conditions framework to analyse the status of integrated care in Greece and to develop an action plan for reform for several reasons. First, the Innovative Care for Chronic Conditions framework has been used as a roadmap for health care system redesign in many countries worldwide with different health care systems and socio-economic contexts [46]. Second, it has inspired a wide range of interventions addressing therefore the local context and needs [47]. Third, we acknowledge that the reform of the Greek health care system in a relatively short time and under the pressure of a severe economic crisis cannot be blueprint adoption of implemented reforms in other countries. Integration of care occurred gradually and in long term in the Western European countries. Thus, best practices in these countries should inspire Greek reforms rather than dictate them. Thus, the building-blocks approach of the Innovative Care for Chronic Conditions framework provides us with flexibility to address the status of the Greek health care and draft an action plan towards integration of care. Last, there is evidence that the Innovative Care for Chronic Conditions framework has the potential to integrate successfully chronic care [46].

The World Health Organization reported a list of challenges at micro-, meso- and macro-level that most health care systems are facing to tackle chronic diseases [22]. We used this list as guidance to search the literature about the failures of the Greek health care system to address chronic diseases. Further, we used published studies and personal experience to provide successful examples for each building block of the Innovative Care for Chronic Conditions framework from other European countries to develop an action plan.

## Current failure to address chronic diseases

### Patient interaction problems (micro-level)

#### Failure to empower patients and their families

The information of chronic patients in Greece about their conditions, expected course and complications, and effective strategies to prevent and manage them varies considerably across patient's socio-economic background and residential area. Chronic patients do not receive adequate information and education about their treatment and they feel that they save time and money by going directly to specialists [48]. This preference is strengthened by the increased co-payments in primary care, which reduces their motivation to participate actively in proactive treatment based in primary care. In the pharmaceutical sector, co-payments increased during the economic crisis by 35%, introducing at the same time a great inequality in the distribution of co-payment burden among the socio-economic groups. The poor spent disproportionally a much higher segment of their income on pharmaceutical care in comparison to richer income classes. The corresponding inter-decile ratio is 4.6. Currently, there are no interventions such as self-monitoring tools and support of self-management skills to prepare chronic patients with behavioural skills to manage their conditions at home [49]. In addition, Greece is ranked as one of the last European Union countries with internet access, and from those citizens with access, only 12% (one of the lowest percentages in Europe) use it to obtain information about health.

Family members of chronic patients are overburdened informal caregivers with low income, without or inadequate pension, low perceived quality of life, feeling heavily burdened and experiencing negative impacts from their caring work [50,51]. Thus, their capacity and motivation to support chronic patients to become self-managers are seriously questioned.

### Problems with health care organisation and its links to the community (meso-level)

#### Absence of continuity and coordination of care

There is virtually no continuity of care for patients with chronic disease by the same care team over time for three main reasons. The first is the high fragmentation and practise variation between the Greek National Health System, the Social Insurance Fund and private primary care practices and hospitals. Even within the Greek National Health System or Social Insurance Funds, the provision of primary and secondary care is fragmented. There is also practise variation between urban and rural provisions of primary care [17]. The second reason lies on the absence of sufficient information systems within primary care and between primary and secondary care. There are no medical records and information exchange between specialists and general physicians is lacking [48]. The proportion of doctors without medical records at hand at a patient visit was the highest among 34 countries [52].

Similarly, coordination of care is severely hampered by the lack of information exchange between health care providers and the absence of a referral system. Greek citizens may seek specialist care without prior consultation with the general physician and go directly to the emergency departments, as the charges for emergency care are low. Visits to medical specialists without referral also have financial implications in terms of out-of-pocket payments [53]. There is also uncontrollable patient flow across regions that leads to many different pathways to care used next to and after each other without coordination [48]. Coordination of primary care is also restricted by the existence of different organisational and administrative structures with insufficient staff and equipment [50].

#### Failure to address prevention

Greece devotes only 0.5% of the health budget on prevention which is the lowest share in comparison to the rest of the European Countries. Although several efforts have focused on preventive action, prevention and health promotion services in the community are limited [17]. Greek general physicians stated to have difficulties in carrying-out prevention and health promotion activities [54]. For the period 2008–2012, a relative large number of health promotion initiatives have been established in the areas of cardiovascular disease, cancer, obesity, nutrition, oral health, and maternal and child health. A smoking ban in public places also has been implemented [55]. However, these initiatives were not effectively implemented and their impact on public health is dubious. Furthermore, the treatment of chronic diseases is highly medicalised with little room for behavioural modification interventions for patients and health care professionals. In addition, rural health centres play a limited role in primary and secondary prevention. This approach is passive not community-oriented, targeting only patients who visit the clinic [48]. Therefore, there is lack of health promotion programmes to inform effectively population and patients about chronic diseases [56]. This lack may be reflected to the high smoking rate, low consumption of fruits and vegetables, high child obesity rates and high cerebrovascular disease mortality compared to the Organization for Economic Cooperation and Development 28 average [57].

#### Inadequate skill-mix and asymmetrical health care workforce

The Greek health care system is physician-dominated and conventionally concentrated on specialist care. Hence the production of health services, from an economic point of view, is based on expensive mix of resources leading to inefficiencies and inequalities in the health delivery. There is a shortage of general physicians, and many of the general physician posts in rural primary care centres are filled by specialists in general practice. This is because Greek medical students show a strong tendency towards specialisation. Primary care physicians have lower social prestige, lower salaries and less employment opportunities compared to specialists. In addition, there is virtually no specialty of general practice in the medical schools (few chairs recognised at three medical schools) [58]. Moreover, tasks and performance between 2-year graduate nurses and 4-year graduate nurses are not different [59]. There is also shortage in nurses especially in primary care despite the high rate of unemployment in the first 5 years after graduation from the nursing school. Greece has the highest number of specialists, the second lowest number of general physicians and the third lowest in nurses among Organization for Economic Cooperation and Development countries [50]. There are also great inequalities in geographical distribution of medical professionals. Despite the specialists’ oversupply, Greek hospitals face significant human resource shortages especially in the area of qualified nursing and high-level administrative personnel. This problem is enlarged by the increasing brain drain of skilled health professionals in Greece who move to North Europe, North America and Australia as a response to the uncertain work environment in Greece during the economic crisis. It is estimated that around 7,500 young doctors left Greece to seek employment during the economic crisis.

Greek universities lack curricula of integrated multidisciplinary care while the establishment of multidisciplinary teams is scarce and not centrally facilitated. In the few examples, the care teams are highly segmented between primary and secondary care and are not trained to support patients to become self-managers. Evidently, the lack of effective clinical information systems to provide access to different health care professionals hampers the establishment of multidisciplinary care teams. In addition, decreases in salaries and increases in workload demotivate health care teams [60].

Despite the geographical, professional and specialties unequal distribution of health care workforce, skill-mix change is not in the health policy agenda and medical and nursing school curricula do not address the needs of patients with chronic diseases. Traditionally, care specialists dominate the setting of the health policy agenda and have powerful lobbying in health authorities including the Ministry of Health. The health care governance is highly hierarchical and doctors lack managerial skills [61]. Considering that specialists are rewarded based on volume, this leaves little room to delegate tasks and roles from specialists to general physicians, nurses, health visitors and eventually new professions. Nursing staff in primary care operate within restricted and task-oriented framework and their education preparation has little impact on practice role variations and medical needs [62]. The potential leadership role and level of involvement of Greek nurses and health visitors towards integrated health care have only recently been pursued as a policy topic. Poor research skills and lack of interest and knowledge in evaluating outcomes of care, for the majority of Greek nurses employed in primary care, have resulted in lack of evidence concerning nursing's contributions to service organisation management, cross-boundary working, management of resources and workforce development. Similarly, social workers have been striving to claim a role within primary care settings, with the discipline of social work being undervalued and unappreciated.

It is generally accepted that medical professionalism is facing a crisis enhanced by the presence of under-table payments and the lack of mechanisms to assess doctor's performance. There is a sizable amount of medical underground economy which contributes significantly to inefficiencies and inequity in the access of health resources [63]. Consequently, there are not many health care leaders able to create an environment that values quality along with the concepts of efficiency and equity.

#### Suboptimal allocation of health technologies

Although primary care centres have increased access to primary care in rural areas, their actual performance has fallen short of expectations due to limited and inadequate staffing, outdated biomedical technology and facilities, and lack of financial and managerial autonomy [17,50]. The Ministry of Health is responsible for controlling capital investments in health. Nevertheless, the process for setting priorities and allocating resources is not clear since a planning and allocation formula has not been developed. The attempt during 2001–2004 to formulate and implement the ‘Health and Welfare Map’ as an instrument for matching the needs of the population with health care resources failed to be implemented.

It seems that there is no coherence in the strategy for expenditure on biomedical equipment, and technologies are introduced without standards or formal consideration of needs, without control of appropriateness, quality and quantity, and without performance monitoring of the equipment installed. This has led to an oversupply of medical equipment of magnetic resonance imaging scanners and computerised tomography scanners leading to over-utilisation and increasing inefficiencies in the diagnostic sector. The problem is further aggravated by the lack of proper incentives determining doctors’ behaviour. Based on unpublished and empirical data, doctors have a financial interest in promoting expensive medical technology and, consequently, tests and procedures are overprescribed. However, a new centralised procurement system for health technologies showed indications of rationality and cost-containment [64].

#### Lack of protocols and guidelines

There are no generally accepted and implemented clinical pathways for chronic patients and clinical practise is not based on (evidence-based) guidelines and clinical protocols. Only recently, prescription protocols are being developed to control pharmaceutical expenditure but they do not encompass comprehensive management of chronic diseases. Greek guidelines for primary care physicians and nurses were also developed but they are still not widely used [65].

#### Information systems are not in place

In order to promote information and communication technologies in Greece, an Operational Programme for the Information Society was formulated in the context of the 3rd Community Support Framework. One of the strategic objectives of the Programme for the period 2000–2006 (which was achieved in 2009) was enhancing citizens’ quality of life through actions to improve the services offered through integrated information and communication technologies in a range of critical sectors, including health. Several pioneering activities took place but they were primarily pilot projects without continuity and they were not integrated into a coherent governmental policy. Before the elections of 2009, the Ministry of Health and Social Solidarity considered a proposal to issue an electronic patient identity card to every insured person. However, this proposal remained on paper and no steps were taken to implement it. Current results on electronic patient record based on episode care were very disappointing and primary care physicians appear to be reluctant in working with electronic patient record systems. This is partially because the concept of the episode of care is still not in operation in primary health care despite its early introduction in Greece [66]. In addition, current modern technologies are excluded from the policy agenda [62]. A recently introduced e-prescription system obliges physicians to prescribe drugs via an electronic system. Almost 90% of the physicians comply with e-prescribing but lack of computer equipment in primary care centres causes additional administrative burden. Only a limited proportion (17%) of the primary care physicians keep medical records in an electronic form [50]. Even physicians who keep electronic records, do not use a uniform system [48].

### Policy problems (macro-level)

#### Legislative framework is lacking

Despite some positive initiatives [67], the bold attempt towards health care reform and integration of regional health and welfare services made by the Legislative Act of 2001 has been negated or inactivated by recent legislation. Recently, a smoking ban in public places has also been implemented conforming to European Union legislation [55]. Human rights of patients with chronic diseases are constitutionally protected but restricted access of these patients to care proves otherwise. Furthermore, even for the passed legislation through the government, there have been considerable delays or even postponements in the implementation process of the anticipated reforms.

#### Limited leadership and advocacy

The policy reforms taken in the last decade in health care lacked political inspiration and were primarily targeted to contain costs [55,68]. The scientific society in Greece provided evidence and suggestions to reform the health care system towards integrated care. However, none of this evidence was taken into account by policy makers when designing reforms [17,62]. Policy makers were driven predominantly by political interest, ‘clientalistic policies’ and lack leadership to inspire, coordinate and motivate stakeholders at the health care and the community level.

**Figure 4.**
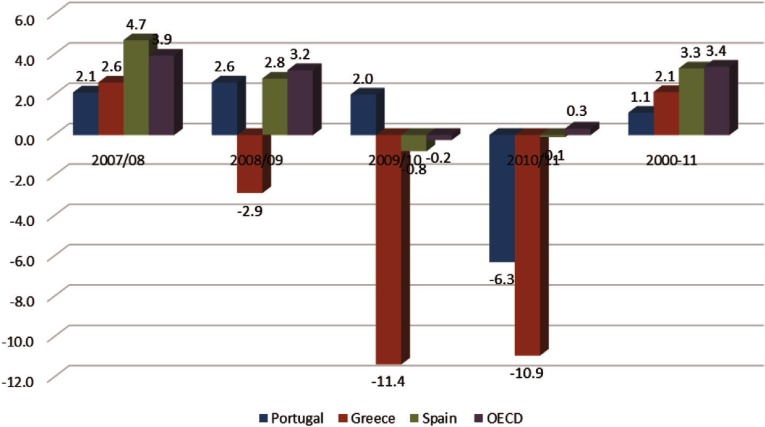
Annual rate of growth of health expenditure 2007–2011. Source: Health at a Glance 2014 [70]

There is no comprehensively designed action plan to reform health care effectively. Most of the reforms after the economic crisis aimed to reduce health care utilisation and publicly financed health care, denoting that the first priorities of the health authorities were to reduce health care budget in a short time. The speed and magnitude of the budget cuts in health care constraint the capacity of the health care system to respond to the increasing health needs. Studies showed that the segmented cost-containment policies reduced public health spending at the expense of access to health care and quality of care [55,68,69]. Figure 4 presents the annual rate of growth of per capita health expenditure before the crisis and during the initial stage of crisis in some ‘memorandum and peripheral’ Eurozone countries as well as across all Organization for Economic Cooperation and Development countries. Before crisis all countries enjoyed a positive increase in their per capita health expenditure ranging from 2.1% in Portugal to 4.7% in Spain. The effects of the crisis are more apparent in Greece, where a reduction of 25.2% is recorded for Greece and only 0.4% in Spain and 6.3% in Portugal.

The reduction in health expenditure was accompanied with increasing inequalities in the access and utilisation of health services. Greece presents the highest inequalities in the access and utilisation of health services in comparison to the rest of ‘Memorandum and Peripheral’ Eurozone countries. The Gini coefficient (which is a measure of inequality ranging from 0 = perfect equality to 1 = perfect inequality) in Greece was the highest (G = 0.295) among all the European Countries (European Union average G = 0.22) in 2014 [71].

Financing decisions are traditionally not based on the principles of equity and effectiveness. Although health care is proclaimed a free public good, high out-of-pocket payments obstruct equity in health. This means that patients with chronic diseases do not have equal access to health care services. Private expenditure is estimated at 37% of which 40% in under-table payment. In addition, 35% of outpatient visits was paid out-of-pocket to private physicians [48].

#### Financing systems are fragmented

Financing of health care is highly segmented. First, a state budget (financed by direct and indirect tax revenues) covers administration expenditures, public hospitals and health centres outside the main urban centres. Second, a large social insurance fund (the National Organization of Healthcare Services) (financed by contributions of employees and employers) runs its own health care units (similar to health maintenance organisations) in urban areas and covers 70% of the population. Third, private expenses including out-of-pocket payments and private insurance schemes that finance a private delivery system, consisting of private hospitals, diagnostic centres and private physicians, most of whom have contracts with the National Organization of Healthcare Services.

Moreover, community involvement, housing, social care, education, welfare and other factors that can promote health are not linked to health care budget in anyway. Community involvement or engagement is not currently a key issue in the Greek health policy, although the subject has received a prompt attention some years ago. Housing, social and welfare care are beyond the scope of the funded activities by the health budget. Therefore, financing of important integrated care services are not linked under a unified system of social accounts.

#### Provider incentives are misaligned

Furthermore, primary care physicians are paid on a salary or a fee-for-service basis. These payment schemes lack specific incentives to stimulate multidisciplinary collaboration and improve the quality of chronic care [23,72]. A salary fails to stimulate integration of care because there are potential incentives to accept only healthy patients (cream skimming) and to refer complex cases to more costly secondary services (dumping). Fee-for-service basis, on the other hand, generates an incentive to provide as many refundable services as possible. While fee-for-service basis reduces the incentive to avoid the chronically ill, there is little incentive for providers to provide high quality of care and adequately address the needs of patients with chronic diseases [26]. Consequently, these traditional payment schemes are unable to facilitate integration and high quality of chronic care [3]. Hospitals are reimbursed via fixed budgets, which are based on historical data, and per-diem fees. Under these payment schemes, hospitals focus on volumes of health care services and not on the provided quality. Therefore, there are no financial incentives to encourage the implementation of innovative care strategies neither the improvement in care quality. The introduction of the Greek Diagnostic Related Groups in 2011 to reimburse hospitals appeared to have reduced length of stay, improved medical and administrative involvement in health management and increased marginally the efficiency of resources. However, this payment scheme encountered a number of problems and its successful implementation is yet to come [73,74]. Finally, there is total absence of home care meaning that services, which could be provided at home by social workers and nurses, are provided at health centres or general physician practices and hospitals increasing, therefore, the financial and capacity constraints in the health care system.

#### Partnerships

Although partnerships among government sectors (e.g., health, social protection, labour, education and agriculture) can potentially influence health and chronic diseases, there is currently absence of structural and coordinated partnerships in Greece. Illustrative is that the health sector is still beside the regional health authorities contrary to other sectors that they are beside the administrative regional authorities. Similarly, there are no connections between regional, municipal and community entities. The cooperation between the public and private health care providers is financially driven without focusing on a common strategy to address the needs of patients with chronic diseases.

#### Governments are not investing wisely

There is lack of an economic evaluation framework to prioritise investments in health care. The financing of health services is based on ‘historical budgets’ (considering only the previous years’ budgets) and does not take into account any performance indicators, outcomes or efficiency criteria. Investing in medical technology is based on the individual interests of politicians, hospital managers and physicians. Thus, many resources have been wasted due to lack of accountability and governments have being investing irrationally during the last decades.

Health Technology Assessment has been widely adopted across Europe with Greece being the only exception. There is no reimbursement (or else Health Technology Assessment) agency to advice decision makers to invest in cost-effective medical interventions and health services. Some proposals in the early 2000s to introduce a health technology assessment system was rejected both by the government and the pharmaceutical companies as an ‘additional hurdle’ to decision-making [75].

#### Standards and monitoring are insufficient

**Figure 5.**
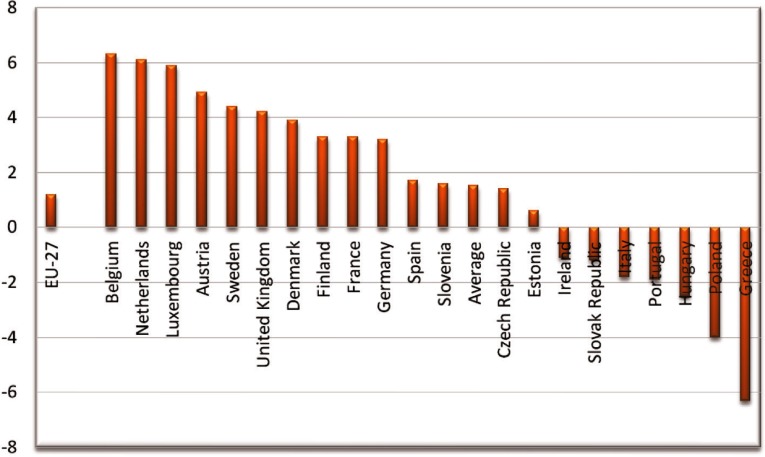
Patient satisfaction with the provision of health services. Source: OECD 2014 Society at a Glance [70]

There is no information about quality of care for several reasons. Quality assurance measurements are not established and indicators to assess the performance of health care providers on processes and outcomes are completely absent. Measures relevant to patient safety in both primary and hospital setting are non-existent [52]. There is lack of patient lists, which in combination with absent personal continuity makes the performance assessment hardly any possible. Since financial incentives are barely focused on the quality of the provided services, there is extensive utilisation of physician consultations, laboratory tests and expensive medical technologies, which is disproportional to the medical needs. At the same time, patient satisfaction remains at one of the lowest levels in Europe (see Figure 5) [50].

## Action plan towards integration of care

### Support a paradigm shift and manage the political environment

Visionary and committed policy makers are essential in creating the momentum for reform towards integrated care [76] and putting integration of care for chronic diseases at the top of the health policy agenda. They also have to be determined and consistent in putting forward the reform, as well as to be persuasive to other health care stakeholders about their devotion to its successful implementation [77,78]. It is necessary to start constructive discussions between health care partners about the need to reform the Greek health care system towards integrated care. Lessons from England highlight the importance of involving care providers in the design of a new care model [79,80]. Evidently, a broader involvement of health care partners enables the smoother reform [81]. Therefore, policy makers, health care authorities, health care providers, social funds and patient organisations should contribute to these discussions by providing explicit views and plans for reform. Understanding the institutional context helps to identify dominant problems of chronic care and find the best solutions [82]. Specific and detailed information on the problem of chronic diseases in Greece should be assembled to motivate all stakeholders to tackle collectively the growing burden of chronic diseases. This is necessary, as a supportive local context was a common precondition for success of integrated care among the Organization for Economic Cooperation and Development countries [83]. A result of this process could be the development of a national policy framework for integrated care to describe how national barriers could be overcome and how local areas could use existing structures. Drafting such a document could be inspired by the ‘Integrated Care and Support: Our Shared Commitment’ issued by a new National Collaborative of key government agencies and non-governmental organisations in England [84].

Furthermore, a thorough and systematic review of existing cost-effective strategies to manage them within and outside the country should be performed to identify models of integrated care suitable for adaptation in Greece (see for inspiration [85]). Similar to many European counties [86], local pilot and demonstration projects of innovative care models could facilitate this adaptation, provide evidence about their cost-effectiveness, and prepare the implementation of new models nationwide. The Netherlands and England facilitated ‘pioneers’ to encourage bottom-up innovation and stimulate local experimentation in a way that avoids a national ‘one-size-fits-all’ approach.

Media could be used as a forum for educating public via publicity, advertising and regular programming, and marketing strategies could persuade the population to think differently about chronic diseases. Powerful and credible voices could be used to spread the message about the growing burden of chronic diseases and the existence of effective strategies for managing them in awareness and education campaigns.

### Build integrated health care

The degree of integration depends on the use of evidence-based guidelines because they standardise care and decision support across different care providers and sites [87]. Similar to other European countries, multidisciplinary guidelines and protocols should be developed in Greece to standardise care and facilitate auditing of delivered care. The development of such guidelines should stem from the consensus between health care professionals, patient organisations and payers (i.e., social insurance funds and government in Greece). Care coordination could be further enhanced by introduction of a gate-keeping system as in most European countries [86]. This system empowers case management and improves care continuity. In England, for example, general physicians working with their wider primary care team of nurses and social workers enable the role of case managers and gate keepers [19]. In other countries, nurses have taken up effectively the role of case manager [88]. Moreover, clinical pathways should be developed to improve coordination and continuity of care as well as patient centeredness [87]. Specifying care coordinators is essential in designing effective clinical pathways [76]. Care groups in the Netherlands act as coordinators of care at the local level. Then specific professionals, most often nurses and general physicians, are responsible for coordinating the care for specific groups of patients. Nurse-led strategies (e.g., nurse-led clinics) are increasingly implemented to further improve coordination [86].

Studies from various countries show that effective information and communication technologies systems are essential in functional integration of care [87]. They can support continuity of services by enabling effective communication between health care providers and promote patient-centeredness by facilitating communication and information exchange between health care providers and patients (e.g., follow-up and active self-management). Therefore, patient registries and information systems should be established or upgraded to increase coordination across public and private health care settings, providers and time. Information sharing strategies across health care organisations and communities should be developed to link health care settings via a common information system.

Most European countries with an advanced level of care integration have been working for more than 20 years towards that way [81]. It would be nonsense to expect that Greece could grasp – in terms of integrated care – these countries within 5 or 10 years. Greece could follow the recent attempts in Spain to reform health care system towards integrated care after following successful pilots in the Basque and Catalonia regions [89,90]. Therefore, a stepwise approach to care integration is recommended. Literature suggested to focus on one care sector to avoid complexities in the early stages of integrating care [81]. Beginning from integrating primary care services should be considered considering its contribution to the overall health system performance [91] and the positive relation between weak primary care and lack of integrated care policies [81]. It can also be extended to allow primary care teams to access specialist advice and support when needed [91]. Beginning by focusing on single diseases rather than on multi-morbidity might also avoid complexities at the early stage of implementation [92]. A literature review study argued that an organised approach in primary care with easily accessible community health centres located in neighbourhoods seems to be suitable in the current Greek setting [17].

### Align sectorial policies for health

The establishment of a multi-sectorial private/public governing body could advocate for the promotion, prevention and comprehensive management of chronic diseases. This requires links to non-health government sectors that have the potential to influence population health. For example, the involvement of municipalities, schools and sport clubs in the population health management was one of the success factors of the evidently cost-effective German Gesundes Kinzigtal model in Germany [93,94]. A regional approach enables such collaborations [95]. Thus, decentralisation of the Greek health care system is necessary to achieve inter-sectoral flexible governing models at regional level. This could follow the decentralisation to municipalities in Finland with continuous renewal to cover the needs of the population focusing on prevention, health promotion and equitable services. Health centres combine primary care and secondary care to improve accessibility in rural areas [96]. In England, there were recent attempts for decentralisation from the Ministry of Health to the recently established National Health System England [97]. In Greece, integration of welfare and care could be achieved, for instance, by upgrading the function of the Open Centres for the Elderly and integrate them in the care system for elderly. Community-based comprehensive geriatric assessments and early intervention strategies could be performed at the Open Centres for the Elderly similar to many other European countries [19].

With respect to illegal immigration, effective partnership between the health sector and other governmental sectors should be established in meeting the needs of immigrants [98]. Moreover, out-of-pocket payment in Greece is partially related to black market economy and illegal immigration. As a result, collaboration between health care authorities and other governmental sectors could address this relation. For example, private payments to illegal health workers could be tackled by providing appropriate financial incentives to informal caregivers.

### Manage health care personnel more effectively

A common element of the several integrated care models is a multidisciplinary team approach accompanied by skill-mix change [86,87,91]. To achieve this, Greek policy makers should focus on four areas. The first area is to change the capacity planning of health workers to match the needs for care at regional level. This could overcome the understaffing problem that many rural areas are currently facing. Adequate personnel capacity planning requires the implementation of joint committees between the Ministry of Health and Ministry of Education to promote a common understanding of medical education needs. In the Netherlands, The Capacity Body (Capaciteitsorgaan) is an important instrument for forecasting and controlling shortages and oversupply in health workforce. It is the exclusive advisory body on the inflow into all specialised postgraduate training programmes and uses a forecasting model for physicians. The final forecasts are discussed with representatives of the profession, health insurers and medical education sector [99].

The second area includes the adequate education of health care providers. New educational curricula for health and social professionals, both at the undergraduate and graduate level, should be established with the emphasis on multidisciplinary training and collaboration. Inter-professional education should be provided by schools of health sciences, while chronic disease management should be promoted in medical schools focusing on non-pharmacological and patient-behaviour modification treatment. Training programmes supported by mandated continuing education should also be provided across a range of health care workers. Nurses should be adequately trained to meet the needs of people with chronic diseases and to fulfil their new role in chronic care delivery. Specialisation of nurses in specific chronic diseases and public health could further enhance their role in chronic care [100]. Social workers should become actively involved in disease prevention programmes, early intervention and effective use of scarce resources. Further, basic skills training should be provided to health and social workers, who help patients with chronic diseases, via workshops and printed materials. Where there are multipurpose health and social workers, study possibilities of reinforcing their decision-making should be provided via linkages with specialists.

The third area is the promotion of skill-mix change. Evidence from the literature shows that new professionals (e.g., advanced nurse practitioners) and new professional roles (e.g., delegation of tasks from physicians to nurses) is cost-effective, if not cost-saving [88]. Besides nurses, the role of pharmacists should also be expanded in Greece. This is because the high capacity and relatively fair geographical distribution of this profession could have an immediate impact on improving chronic care [101]. The role of community pharmacists could be expanded to ensure effective, safe, efficient use of medicine and prevention and management of chronic disease. Based on the experience in Australia, Canada, USA, the Netherlands, Scotland and England, this would require inter-professional collaboration, differentiation of pharmacist services and revised pharmacists’ payment model [102]. A range of new health professions (e.g., self-management counsellors and quality improvement specialists) should be developed to meet the changing health care needs. To support this, training resources should be reallocated in favour of new health professions. Development of new roles for all disciplines, and revision of existing job descriptions, is a critical task that should be undertaken at a legislative, policy and administrative level uniformly for all health regions of Greece. Debating which roles should be assigned to primary care teams or individuals in a multidisciplinary primary care setting should be in line with international experience and supported by a consensus between the involved professions.

The last area concerns the successful implementation of multidisciplinary teams by providing appropriate incentives and legal authorities and responsibilities [103]. Transfer of resources and responsibilities to regional health authorities could facilitate a bottom-up implementation of effective multidisciplinary teams depending on the available personnel and needs of the local population. Regional health authorities could provide training courses and workshops and coordinate the establishment of the teams similar to Denmark [104]. A successful skill-mix change should be supported by information and communication technologies to standardise work processes and to avoid dissatisfaction and distrust between providers [105]. Regional health authorities could facilitate the development of effective information and communication technologies systems in that respect. Appropriate management in health care institutes should facilitate change organisational culture.

### Centre care on the patient and family

Patient centeredness can be achieved through establishing shared decision-making, offering self-management support, and involving patient's family [87]. Shared decision-making between patients and providers is reported frequently in the literature and is followed by self-management support in the form of help for patients and their families to manage their chronic diseases [91]. Self-management support and patient education aiming to change patient's behaviour and lifestyle is a common denominator among all approaches of integrated care in Europe [49,86]. Education could be provided from individual to group sessions and from face-to-face to digital learning programmes depending on the health care capacities and patient preferences. Tele-health and tele-care could be used where cost-effective in terms of geography and disease area, while to enhance patient choice and engagement [106,107].

The role of families and other informal caregivers is crucial for the success of implementing integrated care and the focus should be on enhancing personal networks and the dynamics of the Greek family [108]. However, similar to other Mediterranean countries with prevailing family ethics, family care in Greece is provided only as informal care. This has a negative impact on the health and financial status of informal care providers. To tackle the increasing burden of informal care giving and to support the role of informal caregivers in integrating chronic care, Greece should follow Western European countries that have provided a range of initiatives from cash benefits (in the Netherlands, England) and pension grants (Germany) to training and information (Nordic countries) [19]. These initiatives could be supported by family-centred general practice, which would be included in the educational curricula.

Further, voluntary and community groups as well as non-governmental organisations should be supported to get involved in the provision of care for chronic diseases [109]. Compassionate care should also be part of effective care provision. The roles of these organisations should be supported in policy-making and service planning. Employers should also be informed about chronic diseases management and should take steps to support prevention and self-management in the workplace.

### Emphasise prevention

Strong primary care focusing on prevention and population health is a main characteristic of a high-performing health care system [91]. Countries with strong primary care, such as the Netherlands and UK, emphasise integrated approaches to link health and health promotion efforts to disease management and self-management support [9]. Greece could follow the same approach by ensuring that the prevention of chronic diseases is addressed in primary health care visits, monitoring risk factors and identifying persons at risk for developing chronic conditions, and assisting primary care providers through education and tools to put prevention in the spotlight of every patient encounter.

Further, population-based prevention activities should be promoted at regional and national level in Greece. For example, evidence-based breast and cervical cancer screening programmes organised at national level and comprehensive geriatric assessment of elderly organised at regional level tailored to the needs of the elderly and the local health care setting (which varies from remote islands and rural areas to highly dense urban areas). Moreover, regulation and legislation that curbs the marketing of public health risks (e.g., tobacco and alcohol) should be enacted. Ban alcohol and tobacco selling to young people and actual implementation of smoking in public places and restaurants are some examples of legislative interventions to improve population health.

### Ensure adequate and sustainable financial support for innovative care

Universal coverage is a prerequisite of a high-performing chronic care systems in which people with chronic conditions have access to high care quality [91]. Considering the high out-of-pocket payments in Greece, this issue should be the first to be addressed in the health policy agenda. Discouraging population to access primary care providers jeopardises any effort to promote prevention, care continuity and self-management.

On the providers side, several payment schemes have been implemented in Europe to incentivise integration of care [110]. Similar to the Netherlands, Austria and Germany, Greece could incentivise care coordination to overcome fragmentation within primary care and between primary and secondary care [111]. Establishing groups to contract and commission integrated care for people with chronic diseases similar to the Netherlands and England could strengthen coordination. These groups could negotiate with health care payers and providers retrospective fixed payments for the care that chronic patients need in a year or even longer periods following the successful Gesundes Kinzigtal model in Germany [95]. Similar to other European countries, elements of competition between care providers could enhance quality of care. The introduction of Quality and Outcomes Framework in England and bundled payments in the Netherlands have introduced elements of competition between contracted health care providers by reimbursing them based on performance indicators. Key performance indicators for processes (including prevention) could be implemented in Greece to help keep new integrated care models sustainable in time similar to the virtual wards pilots in England where multidisciplinary processes were evaluated based on team performance [79]. To begin with, primary care providers in Greece could be reimbursed based on a three-tier system similar to a newly introduced payment reform in the Netherlands. Under this payment scheme, general physicians could be reimbursed 75% on (risk-adjusted) capitation basis, 15% for multidisciplinary collaboration and 10% based on performance indicators. Additional financial resources to reward providers could be generated by tighter control of expensive medical technology due to either overcapacity of necessary technologies or reimbursement of cost-inefficient technologies [62]. To achieve this, a Health Technology Assessment agency should be effectively established to support rational investing in health care.

Further integration could be stimulated by pooling health and social care budgets similar to England [97]. Patient's control of the care process could be enhanced by providing individual budgets to people with chronic diseases to purchase home care similar to the Netherlands and England [19].

Any form of financial agreement to stimulate integrated care should be subject of policy evaluation by measuring outcomes at national (e.g., expenditure), organisational (quality of care,) and patient (e.g., clinical) levels for continuous improvement.

## Discussion

This study showed that chronic conditions are poorly addressed in Greece and integrated care is in embryonic stage. Taking this into account and considering the increasing health care expenditure and tight health budget, this the right time to introduce reforms in the Greek health care system to integrate care for chronic diseases [62]. Optimists would argue that Greece should see the crisis as a blessing to reform care rather than as disaster of health care [14]. However, even they would agree that this is not an easy task.

Greece needs visionary politicians with willingness and commitment to make fundamental changes and to be confronted with long-standing and well-established interests against the required reforms. They and their electorate need to understand that health is a public good that is above the political interests of any political party. Immediate actions should be taken towards sustainable integrated care, which should not be seen as a cost containment policy to restrain health expenditure growth in the short term. Many European countries experimenting more than 20 years to successfully integrated care [81] and evidence from Organization for Economic Cooperation and Development countries shows no economies of scale and economies of scope in the short term [83]. Integrated care should be seen as a reform for development with high return in the long term rather than another austerity measurement to keep health care budgets tight. During times of heavy crisis, prioritising for efficient resource allocation would generate the potentials to make the appropriate reforms towards integrated care while staying within given governmental budgets. Therefore, efficiency and productivity should be the drivers to achieve integrated care. Measuring improvements in these aspects at system level based on predefined target measurements are crucial to the successful implementation of a dynamic policy reform [112].

Considering the high opposition to evidence-based reforms, Greek policy makers should better design bottom-up policies with effective governmental steering via financial reforms and legislation that allows for stimulated innovation. Based on the early experience with integrated care in the Netherlands, blueprint policies create rigidities and highly political opposition [82]. At the same time, the Greek medical and administrative personnel should present the appropriate resilience and willingness to adopt the required structural reforms for integrating care for chronic diseases.

Primary care might be seen as the key component and starting point for integrating care in Greece. It is considered the vehicle towards coordinating chronic care, promoting prevention and public health, facilitating skill-mix change, accommodating e-health technologies and controlling unnecessary hospital expenditure. There is a pile of literature with different initiatives to build up a strong primary care with the most recent report highlighting the building blocks on how policy makers can achieve that [113]. Literature along with knowledge platforms, such as the European Forum for Primary Care and the International Foundation for Integrated Care, should support with evidence the steps to be taken in reforming the Greek primary care. Establishing a committee of national and international experts in integrating primary care could be used as a spearhead to designing the necessary reforms.

Moreover, reforms with a theoretical basis that have been operationalised elsewhere and proved successful might be the initial step in orienting policy-making to reform the Greek health care system. Disease management programmes were developed based on the Chronic Care Model and implemented in the Netherlands, Austria, Germany, France, Denmark and Hungary. Choosing for integrating care through the implementation of disease management programmes might be an attractive starting point for the designers of health care reforms in Greece. Experience from these countries can be used to avoid barriers in their implementation and accelerate their implementation by stepping on existing evidence. However, we do acknowledge that there is a current shift in many countries towards adopting wider models of health and social care integration or population health management to deal with the non-medical side of care [114]. These models could support independent living or healthy lifestyles in the recognition that disease management alone is not enough for the most complex patients such as patients with multi-morbidity. However, the implementation of such models would require more time, infrastructure, financial resources, willingness for radical reforms across pubic sectors, inter-professional education in the academic institutes, proper organisational culture and structures of health and social services, all of which are currently scarce in Greece.

No matter what the chosen mean to integrated care in Greece, the focus of the reforms should be on people centeredness. The health care system should be redesigned to serve citizens with high-quality care and to respond to their preferences. It has to respond to their needs and preferences in human and holistic ways as the one-size-fits-all approach is outdated, inefficient and highly inadequate to deliver care to people with chronic diseases. The World Health Organization has recently published an interim report on people centeredness and integrated care calling for policy makers worldwide to achieve integrated care by focusing on patient-centeredness. The Greek Ministry of Health should not be an exemption in doing so.

Furthermore, research should play a central role in reforming Greece's health care system towards integrated care. Designers of policy reforms should take into consideration existing evidence from the literature and the pioneering attempts of introducing elements of integrated care in Greece [115]. Evidence of what works, how it works and for whom it works should be created by allocating research funds to the academic and clinical community. Research guidelines should be issued to drive research in providing conclusive evidence on topics with high policy implication impact.

## Conclusion

Greek policy makers have to realise that this is the opportunity to make substantial reforms in chronic care. Failing to reform towards integrated care would lead to the significant risk of the collapse of the Greek health care system with all associated negative consequences. The action plan provided in this paper could support policy makers to make the first serious step to face this challenge. The details and specifications (e.g., prioritisation, coordination, control and time planning) of the action plan can only be decided by Greek policy makers in close cooperation with other health and social care partners. This is the appropriate time for doing so.

## Figures and Tables

**Figure 3.**
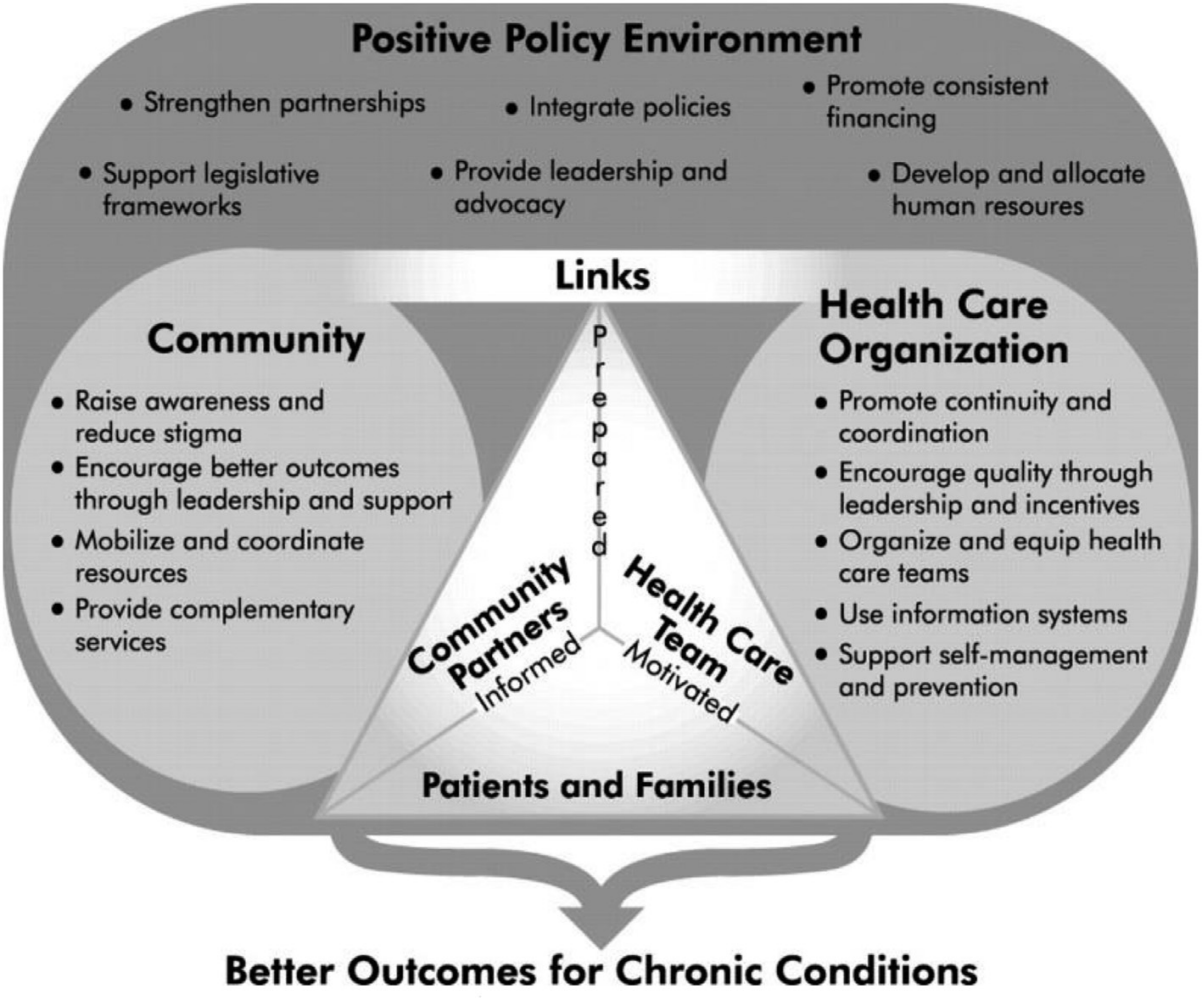
The Innovative Care for Chronic Conditions Framework. Source: Innovative Care for Chronic Conditions: Building Blocks for Action, Geneva, Switzerland, World Health Organization, 2002, pp. 65). Copyright 2002, World Health Organization. Reprinted with permission. [22]
